# Quenching of Protein Fluorescence by Fullerenol C_60_(OH)_36_ Nanoparticles

**DOI:** 10.3390/ijms232012382

**Published:** 2022-10-16

**Authors:** Anna Lichota, Mariusz Szabelski, Anita Krokosz

**Affiliations:** 1 Department of Molecular Biophysics, Faculty of Biology and Environmental Protection, University of Lodz, 90-236 Lodz, Poland; 2 Department of Physics and Biophysics, Faculty of Food Science, University of Warmia and Mazury in Olsztyn, 10-719 Olsztyn, Poland; 3 Department of Biophysics of Environmental Pollution, Faculty of Biology and Environmental Protection, University of Lodz, 90-236 Lodz, Poland

**Keywords:** fullerenol, human serum albumin, alcohol dehydrogenase, time-resolved fluorescence spectroscopy

## Abstract

The effect of the interaction between fullerenol C_60_(OH)_36_ (FUL) and alcohol dehydrogenase (ADH) from *Saccharomyces cerevisiae* and human serum albumin (HSA) was studied by absorption spectroscopy, fluorescence spectroscopy, and time-resolved fluorescence spectroscopy. As shown in the study, the fluorescence intensities of ADH and HSA at excitation wavelengths λ_ex_ = 280 nm (Trp, Tyr) and λ_ex_ = 295 nm (Trp) are decreased with the increase in the FUL concentration. The results of time-resolved measurements indicate that both quenching mechanisms, dynamic and static, are present. The binding constant K_b_ and the number of binding sites were obtained for HSA and ADH. Thus, the results indicated the formation of FUL complexes and proteins. However, the binding of FUL to HSA is much stronger than that of ADH. The transfer of energy from the protein to FUL was also proved.

## 1. Introduction

The first and most well-known fullerene, C_60_ (buckminsterfullerene), has a hydrophobic character with a tendency to form aggregates in water solutions. The solubility of fullerene is less than 10^−9^ mg/L in water [1,2,3]. Fullerenes possess high electron affinity and do not require a large amount of energy in the electron transfer process [4]. The chemical constitution, i.e., the presence of rigid or saturated hydrocarbon bridges, performs an important role in the electronic coupling between the donor and the acceptor [5]. Fullerene and its derivatives are a photosensitive and luminescent material [6]. In the excited state, fullerene C_60_ displays perfect electron acceptor-donor properties [7]. The photoactivation of fullerenes generates singlet oxygen and other reactive oxygen species in the treated cells and cancers [8].

Therefore, fullerene nanoparticles began to be modified to fullerenols (fullerols, polyhydroxylated fullerenes) to enhance their solubility and decrease their toxicity in in vitro and in vivo systems [9,10,11]. Fullerenols are currently the most studied class of water soluble derivatives of fullerenes. Due to their chemical structure, fullerenols are promising drug carriers and antioxidants [12]. As indicated by the analysis of the available literature [13,14,15], the quality and quantity of substituents attached to the fullerene core is of key importance to the biological properties of the obtained fullerene derivatives. The antioxidant activity of fullerenols is associated with the absorption of electrons and oxygen radical scavenging [9,10,11]. The water soluble C_60_ derivatives can quench a very wide range of fluorescence dyes, which is a great advantage in biological research [16]. Additionally, Andreoni et al. [17] confirmed that fullerenes and their derivatives have very low fluorescence quantum yields, which are lost with increasing aggregation. Thanks to these properties, monitoring of fullerenes and their water soluble derivatives is possible. The studies have demonstrated that they have clinical applications as carriers to transport drugs across the brain and ocular barriers [18]. In the work of Nepomnyashchaya et al., it is also shown that fullerenol can be used as a probing substance for the analysis of structural changes in proteins [19].

Human serum albumin allows for the transport of fatty acids, hormones, drugs, or metabolites and the disposal of endogenous and exogenous compounds, and, additionally, it is responsible for osmotic blood pressure [20,21,22]. The binding of drugs to HSA depends on many factors (drug solubility, toxicity, susceptibility to oxidation, in vivo half-life, etc.) [22,23]. The structure of HSA consists of three homologous α-helical domains (I-III) and each domain is further divided Into subdomains A and B [24,25,26]. The domains, despite having similar structures, have a different affinity for ligands. Two important binding sites on human serum albumin are Sudlow site I and site II, which are located in subdomains IIA and IIIA, respectively [24,25,27]. The protein is formed by 585 amino acid residues containing 17 disulfide bridges and a single tryptophan residue (Trp-214) located in the hydrophobic cavity of site I [26,28]. The binding affinity offered by site I is mainly executed through hydrophobic interactions, whilst the affinity of site II involves a combination of hydrophobic, hydrogen bonding, and electrostatic interactions [29]. In the study by Sharoyko et al., the binding of C_60_(OH)_24_ to HSA showed that the interaction of the fullerenol with HSA occurs through the subdomains IB and IIIA [12]. Studies in mice demonstrated that the fullerenol-doxorubicin conjugate had high antitumor efficacy without the systemic toxicity of free doxorubicin [30]. Furthermore, fullerenol C_60_(OH)_44_ had little influence on the secondary structure of bovine serum albumin (BSA) and γ-globulins. The cytotoxicity tests showed that the presence of proteins attenuated the toxic effect of fullerenol on the human normal gastric epithelial cells of line GES-1 [31].

An important group of enzymes are NAD(P)-dependent oxidoreductases, which occur in virtually all organisms and catalyze the reversible oxidation of primary and secondary alcohols into aldehydes and ketones, respectively. Oxidoreductases include ADH, which has a tetrameric structure in prokaryotes and lower eukaryotes (yeast). Yeast ADH is a tetramer of four identical subunits with 347 amino acid residues each [32]. Each subunit is separated into two domains, one ‘coenzyme-binding’ and one ‘catalytic’. Based on the fact that each individual chain contains one reactive sulfhydryl group and, by binding one atom of zinc and 1 mol of NAD^+^/NADP^+^, it is capable of forming an independent ‘active center’ within the quaternary structure of the active tetramer [33].

As fullerenols can be used as drug carriers and have many useful properties in biological systems, it seems important to determine the interaction and binding of FUL to proteins. Two proteins differing in structure and function were selected to determine FUL binding to HSA and ADH.

Although other studies [31,34,35] investigated the interactions of proteins with fullerene derivatives, none of them analyzed the interactions of highly hydroxylated fullerenol C_60_(OH)_36_. In this study, the ability of fullerenol C_60_(OH)_36_ to bind to proteins (alcohol dehydrogenase from *Saccharomyces cerevisiae* (ADH, EC 1.1.1.1) and human serum albumin (HSA, EC 274-272-6)) was investigated. To study the interactions between fullerenol C_60_(OH)_36_ and proteins, UV-Vis absorption spectroscopy, fluorescence spectroscopy, and time-resolved fluorescence spectroscopy were used.

## 2. Results

### 2.1. Characterization of C_60_(OH)_36_ Aqueous Suspensions

**Figure 1:**
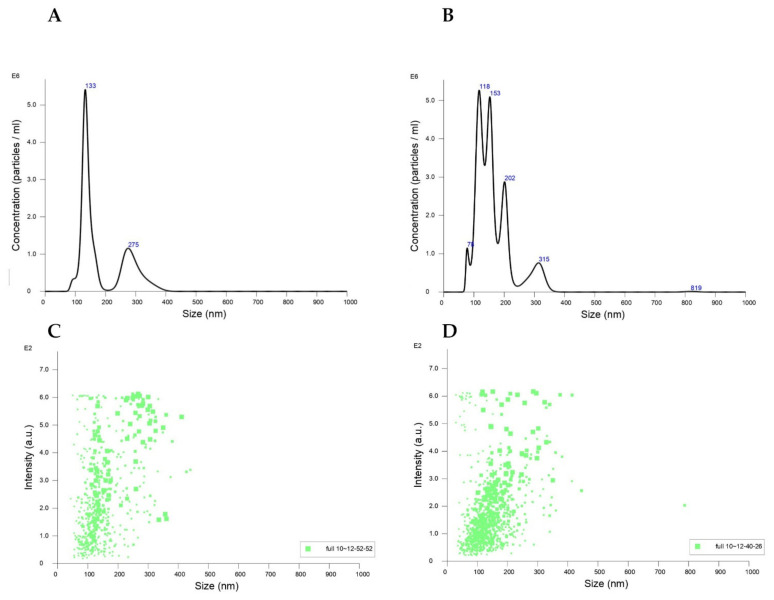
The size distributions of fullerenol (8 μM) in water (**A**,**C**) and 0.02 M phosphate buffer pH 7.4 (**B**,**D**) measured by NTA; (size vs. concentration graph—**A**,**B**; size vs. intensity graph—**C**,**D**). The measurements were carried out at 22.6 °C. The results from NTA were acquired by finite track length adjustment (FTLA). The mean size and SD values of the analyzed particles were obtained by the NTA software.

The nanoparticle tracking analysis (NTA) is a new analytical method for nanoparticles [36]. The NTA method is based on tracing the movement of particles and measuring the flashes of light scattered on the particles, on the basis of which their size is determined. The size distributions as well as the particle concentrations are shown in the graphs (Table 1, Figure 1). The mean diameter of the fullerenol nanoparticles in the aqueous solution was 186.5 ± 76.6 nm, while in the 0.02 M phosphate buffer pH 7.4, it was 164.4 ± 65.6 nm.

### 2.2. Spectral Characteristics of Fullerenol

**Figure 2:**
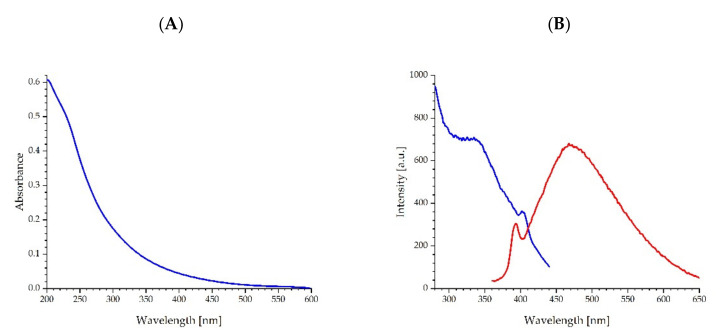
Absorbance spectrum (**A**), excitation, and emission spectra (**B**) of fullerenol (5.36 μM) in phosphate buffer (blue line—observation at λ_ob_ = 470 nm; red line—excitation at λ_ex_ = 340 nm).

**Figure 3:**
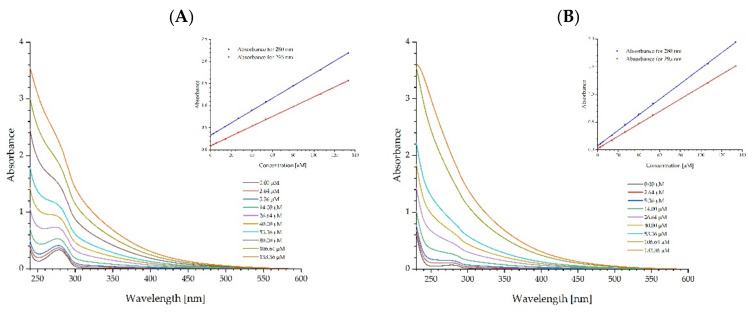
Absorption spectra of ADH (**A**) and HSA (**B**) proteins in solutions containing increasing fullerenol concentrations.

The spectroscopic properties of fullerenol have been investigated. Figure 2A shows the UV-Vis absorption spectrum of fullerenol in phosphate buffer with a slightly visible inflexion in the range of 330–350 nm. This deformation is the FUL absorption band with a maximum of about 340 nm, which is confirmed by the excitation spectrum shown in Figure 2B. Using an excitation wavelength of 340 nm, the fluorescence emission spectrum was measured and shown in Figure 2B. The fluorescence maximum of fullerenol in phosphate buffer is found at a wavelength of 470 nm. To check the interaction of fullerenol with ADH (Figure 3A) and HSA (Figure 3B) proteins, the absorption spectra of solutions containing a constant protein concentration and an increasing concentration of FUL were measured. It was observed that with increasing concentrations of fullerenol, there is only a proportional increase in the absorption of fullerenol but there is no visible change in the protein absorption band. We observe a rectilinear relationship between the FUL absorbance and its concentration for both 280 nm and 295 nm observation wavelengths. Thus, in the concentration range of 2.64 to 133.36 μM of fullerenol, the Beer-Lambert law is fulfilled, which proves FUL stability in the buffer solution and the absence of its aggregation. Table 2 summarizes the molar absorption coefficients at 280 nm for fullerenol, ADH, and HSA in 0.02 M phosphate buffer at a pH of 7.4. Observations of absorption spectra did not provide sufficient data to solely discuss protein-fullerenol process associations in the ground state. For this reason, further fluorescence measurements were performed to assess the possibility of a protein-fullerenol association.

### 2.3. Protein Fluorescence Quenching by Fullerenol

**Figure 4:**
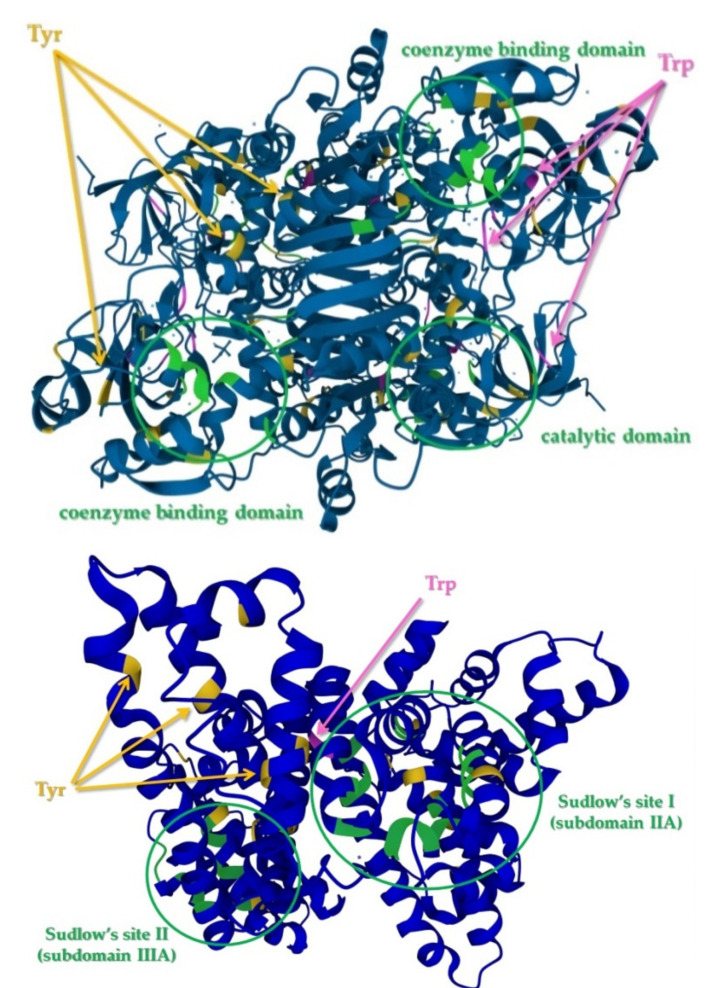
ADH [39] (**top**) and HSA [40] (**bottom**) structures with Trp and Tyr positions indicated. Structures of both proteins are from the PDB—Protein Data Bank.

ADH and HSA owe fluorescence properties to the presence of aromatic amino acids in their structure—tryptophan and tyrosine. ADH contains 5 Trp residues and 14 Tyr residues, whereas HSA contains 1 Trp residue and 18 Tyr residues. In general, the main regions of small molecule binding sites on HSA are located in the hydrophobic cavities in subdomains IIA and IIIA, which are also referred to as Sudlow’s site I and site II. Yeast ADH is a tetramer, and each subunit is separated into two domains, one ‘coenzyme-binding’ and one ‘catalytic’ (Figure 4) [32,33,37,38].

In general, fluorescence quenching refers to any process which decreases the fluorescence signal of the studied object. A different type of molecular interaction can result in quenching, including excited-state reactions, molecular rearrangements, energy transfer, ground-state complex formation, and collisional quenching. As a result of these processes, we will observe perturbation or modification in the fluorescence parameters such as intensity, quantum yield, and/or lifetime. Fluorescence quenching is an important technique for measuring the binding affinity between ligands and proteins, but the interpretation of the results is not simple because different processes can occur at the same time. Moreover, the emission intensity of Tyr and Trp residues located on the protein surface will be strongly decreased in the presence of a quencher. On the other hand, the emission of buried amino acids will be less affected by the quencher and will depend on the distance between the fluorophore and the quencher.

**Figure 5:**
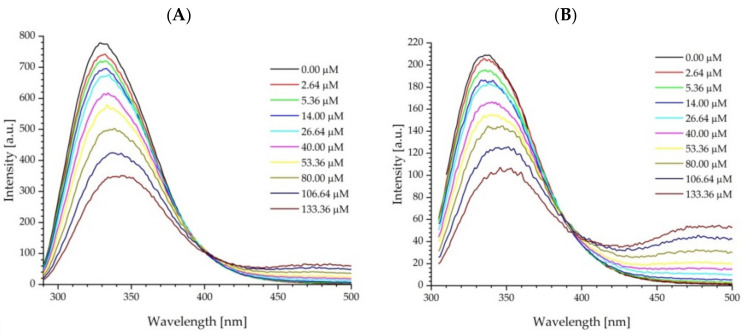
Quenching of ADH fluorescence by fullerenol C_60_(OH)_36_ nanoparticles. The excitation wavelengths were λ_ex_ = 280 nm (excitation of both amino acids Trp, Tyr; (**A**) and λ_ex_ = 295 nm (Trp excitation only; (**B**).

**Figure 6:**
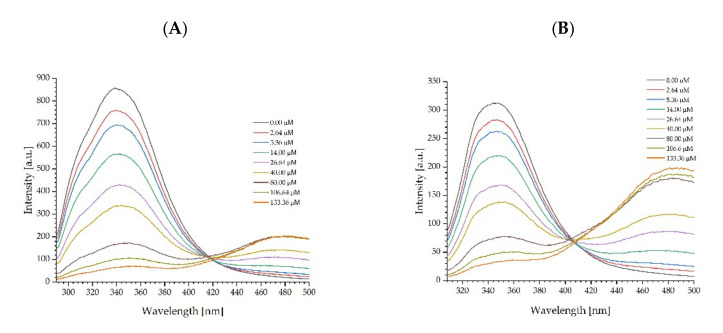
Quenching of HSA fluorescence by fullerenol C_60_(OH)_36_ nanoparticles. The excitation wavelengths were λ_ex_ = 280 nm (excitation of both amino acids Trp, Tyr; (**A**) and λ_ex_ = 295 nm (Trp excitation only; (**B**).

To investigate the binding of fullerenol C_60_(OH)_36_ to ADH (Figure 5) and HSA (Figure 6), fluorescence spectra were recorded. The samples containing 2 μM of protein and an increasing concentration of FUL varying from 0 μM to 133.36 μM were excited with 280 nm and 295 nm wavelengths.

The existence of an overlap between the absorption bands of proteins and fullerenol was noticed. However, the results presented in Figure 3 show that in the concentration range used, fullerenol C_60_(OH)_36_ obeys the Beer-Lambert Law and applying the correction on the effect of the absorber (FUL) allows for the assessment of the protein fluorescence quenching by FUL.

All fluorescence measurements were corrected on the inner filter effects. The corrections of fluorescence were carried out in accordance with the methodology described in detail in the papers [41,42]. As the data show, the fluorescence intensity of ADH and HSA decreased and the protein band redshifted with the increasing concentration of FUL, which indicates that FUL can bind to these proteins. The intrinsic fluorescence maximum of ADH is shifted from 330 nm to 340 nm and from 335 nm to 350 nm for excitation wavelengths 280 nm and 295 nm, respectively. Similar changes were observed for HSA, where the protein band maximum at 340 nm was shifted by 10 nm with an excitation of 280 nm, whereas with an excitation of 295 nm, the shift toward longer wavelengths was bigger and amounted to 20 nm. Furthermore, at a higher fullerenol concentration, a new fluorescence emission can be observed in Figure 5 and 6, initially as a shoulder and, afterwards, as a band with the maximum at the position of the free FUL emission.

**Figure 7:**
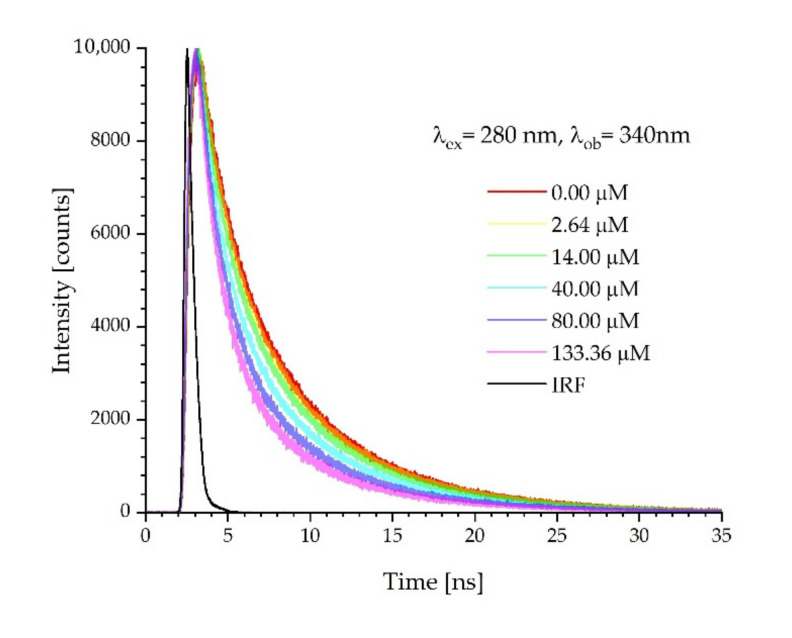
Fluorescence intensity decays of HSA with different FUL concentrations recorded at the emission wavelength, λ_em_ = 340 nm.

The changes in emission spectra are the summarized fluorescence signals originating from processes occurring in both the ground and excited states. The problem of whether quenching processes occur in the excited state can be solved by time-resolved fluorescence measurements. The fluorescence lifetime remains unchanged with the addition of the quencher in static quenching, but changes with the added quencher in dynamic quenching as this is a time-dependent process [43]. Therefore, in this study, fluorescence time decays of titrated proteins were measured at an excitation wavelength of 280 nm. The observation wavelength was dependent on the position of the fluorescence signal maximum in the first sample from the experimental series, and this was 330 nm and 340 nm for ADH and HSA, respectively. Examples of fluorescence decays obtained for HSA are shown in Figure 7. The lifetimes of proteins and their complexes with the FUL in the excited state were computed. The fluorescence decays obtained for all probes were described by a three-exponential function. The results of the lifetime calculations are presented in Table 3.

### 2.4. Mechanism of Fluorescence Quenching by Fullerenol

Quenching occurs through either static or dynamic interaction mechanisms. Both types of interactions require molecular contact between the fluorophore and the quencher. Static quenching occurs in the ground state and a non-fluorescent complex is formed between the fluorophore and the quencher. In the case of collisional quenching, the quencher must diffuse to the fluorophore during the lifetime of the excited state. As a result of contact with the quencher, the fluorophore returns to the ground state without emitting a photon [43]. In both cases, it leads to a decrease in the overall measured intensity of the fluorescence signal, but only the dynamic mechanism reduces the excited lifetime. In this work, we used the Stern–Volmer approach and its modifications to analyze the fluorescence quenching of ADH and HSA. The classical Stern-Volmer equation relates the drop in fluorescence to the concentration of a collisional quencher, as

(1) $$\frac{I_{0}}{I}=\frac{\tau_{0}}{\tau }=1+K_{\mathrm{SV}}\left[Q\right]$$

where I is the fluorescence intensity in the presence of the quencher, I_0_ is the intensity in the absence of the quencher, [Q] is the quencher concentration, and K_SV_ is the Stern-Volmer quenching constant. The Stern-Volmer plot can also be obtained from quenching the fluorescence lifetime and, in this case, lifetimes in the absence (τ_0_) and the presence (τ) of Q are used instead of the fluorescence intensity. The above equation predicts a linear plot of I_0_/I versus [Q] for a homogenously emitting solution. In many cases, the fluorescence quenching is characterized by a nonlinear Stern-Volmer plot. The upward deviation of the plot of I_0_/I against [Q] from linearity may be caused by the simultaneous occurrence of static and dynamic quenching. Additionally, the quencher and the fluorophore do not form a non-fluorescent complex during the static quenching but are in such a close proximity that fluorescence is quenched immediately upon excitation. This mechanism is called sphere-of-action quenching. The following general relation can then be used:

(2) $$\frac{I_{0}}{I}=\left(1+K_{\mathrm{SV}}\left[Q\right]\right)\exp\left(V\left[Q\right]\right)$$

**Figure 8:**
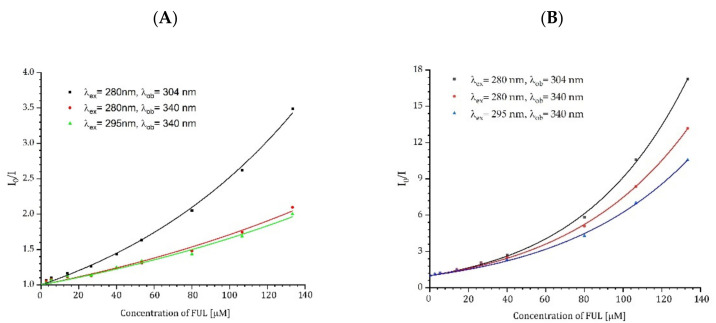
Stern-Volmer plots of ADH (**A**) and HSA (**B**) fluorescence intensity quenching with FUL.

Where V is the static quenching constant in M^−1^. The constant V is directly dependent on the volume V_q_ surrounding the fluorophore (V = V_q_N_a_), which is called the active sphere or quenching sphere. V_q_ is expressed in L and N_a_ is Avogadro’s number. The quencher will interact with the fluorophore only when it is inside the active sphere, otherwise HSA has no effect at all on the fluorophore [43,44,45,46,47]. The sphere-of-action quenching is often found in protein research due to the fact that Tyr and Trp are inside the protein structure and are located in or near the binding site. In our studies, we also observed an upward curve of *I_0_/I* on the quencher concentration (Figure 8). The fluorescence changes were collected for two excitation wavelengths, λ_ex_ = 280 nm and λ_ex_ = 295 nm, and observations were taken at λ_ob_ = 304 nm and λ_ob_ = 340 nm to determine with which amino acids, Tyr and/or Trp, the FUL nanoparticles interacted. The selection of 280 nm as an excitation wavelength resulted in the simultaneous excitation of Tyr and Trp, whereas the use of λ_ex_ = 295 nm excited only Trp. Similarly, by selecting the appropriate observation wavelength, we could record the signal mainly coming from Tyr or Trp and it was λ_ob_ = 304 nm and λ_ob_ = 340 nm, respectively.

**Figure 9:**
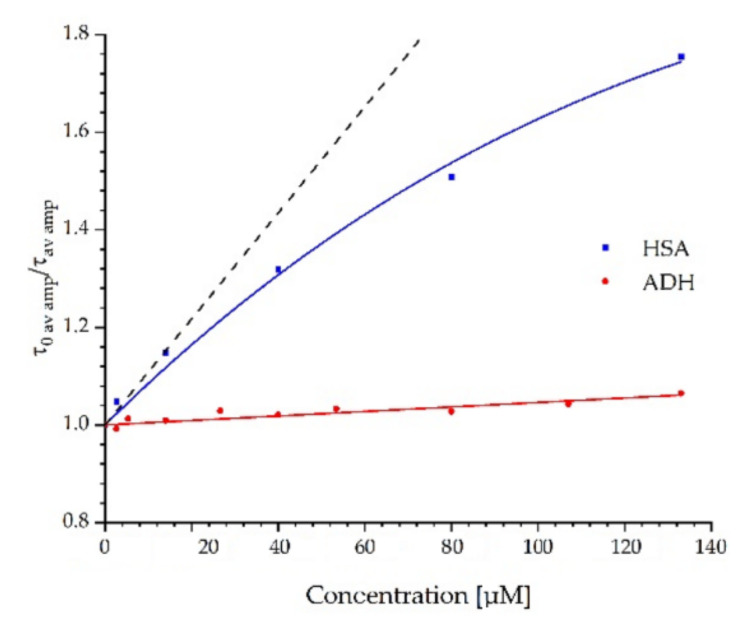
Stern-Volmer plot of the amplitude-weighted lifetimes of ADH and HSA with the FUL recorded at the emission wavelength λ_em_ = 340 nm. The black dotted line–linear fit for the lowest FUL concentrations (up to 13.36 μM).

The effects of the quencher on the fluorescence lifetime of ADH and HSA were also measured, as shown in Figure 9. Changes in the lifetimes confirm the existence of the dynamic quenching mechanism. Due to the three-exponential decay of fluorescence, the amplitude-weighted average lifetime of proteins in the absence and the presence of FUL were used to calculate K_SV_ [48]. Results for ADH were linear and fitted using Equation (1). Unlike ADH, the Stern-Volmer plot for HSA was nonlinear. The downward curve suggests the presence of additional processes between HSA and FUL in the excited state. Using the results for the lowest FUL concentrations, we determined the tangent to the curve and on its basis, we determined K_SV_ using Equation (1).

The results from time-resolved measurements indicate that both quenching mechanisms, dynamic and static, are present. The data obtained from the fluorescence spectra collected during the quenching experiment were fitted to the Stern-Volmer in Equation (2). Equation (2) fitted to the ADH results had no solutions. In this situation, the value of the constant K_SV_ in Equation (2) was taken from time-resolved measurements and set as fixed. Thanks to that, it was possible to calculate the constant for the static quenching process for ADH. The calculated K_SV_ and V values obtained from time-resolved and steady-state measurements are listed in Table 4.

### 2.5. Determination of the Binding Parameters

**Figure 10:**
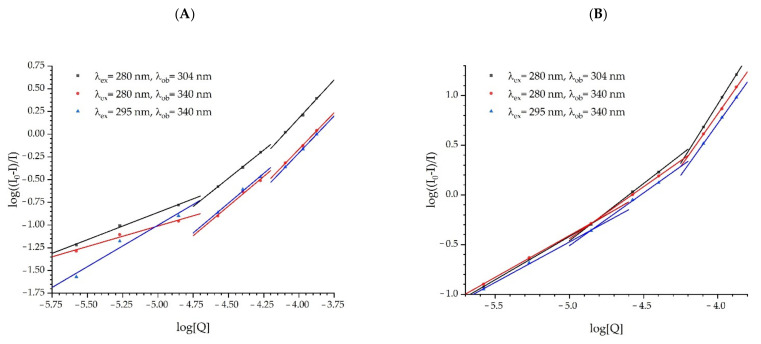
Dependence of log[(I_0_-I)/I] on log[Q] for ADH (**A**) and HSA (**B**) with FUL. Q is a quencher i.e. FUL.

Figure 10 shows the dependence of log[(I_0_-I)/I] on log[Q] for the studied proteins obtained from fluorometric titration. One can see that three data segments are visible for each titration. Data in the segment are linear. This situation indicates the presence of three different protein conformations, which merge into each other with the increasing quencher concentration. Each linear data segment is one protein conformation. To determine the binding constant K_b_ and the number of binding sites n, the obtained results were fitted to the following linear equation [49]:

(3) $$\log\left[\frac{I_{0}-I}{I}\right]={\mathrm{logK}}_{b}+\mathrm{nlog}\left[Q\right]$$

The results of the fitting are presented in Table 5. The K_b_ values for the association of the studied proteins strongly depend on fullerenol concentration. A similar situation was observed in the case of the number of binding sites.

### 2.6. Energy Transfer

**Figure 11:**
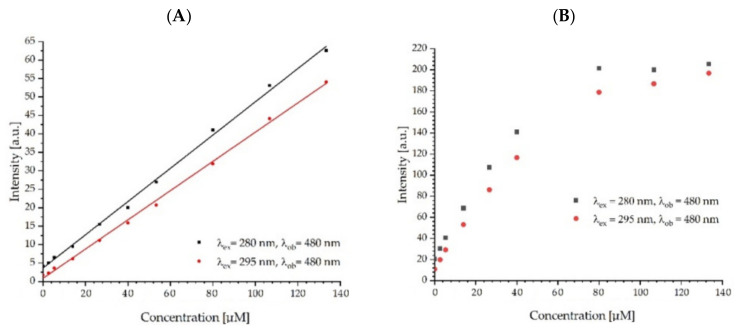
The fluorescence intensity of fullerenol in complexes with ADH (**A**) and HSA (**B**).

Figure 5 and Figure 6 show an additional fluorescence band, which increases with increasing fullerenol concentration. The maximum of this band is around 480 nm, which corresponds to the FUL fluorescence. Figure 11 shows the changes in the fluorescence signal read at the maximum of the long wave emission band (480 nm) at excitations of 280 nm and 295 nm. Changes in the fluorescence intensity of fullerenol are greater in the case of an excitation of 280 nm, which may be caused by the simultaneous interaction of the FUL with Tyr and Trp and the energy transfer from these amino acids to the FUL and/or the occurrence of energy transfer from Tyr to Trp and, further, to FUL. The phenomenon of energy transfer from Tyr to Trp in proteins is well known [43,50]. We observe a linear plot for ADH and a downward curve for HSA. In the case of HSA, for the concentration of the quencher, we observe protein saturation by fullerenol. These observations correspond well with the data presented in Table 5.

Fluorescence decays over time were measured to confirm the energy transfer from the studied proteins to fullerenol. Three types of samples were measured: the FUL in the phosphate buffer, ADH-FUL, and HSA-FUL complexes. The fluorescence signal of fullerenol was collected for 470 nm with excitation wavelengths of 375 nm and 280 nm for the FUL in the buffer and protein-FUL complex, respectively. The calculated lifetimes of FUL are presented in Table 6. The amplitude-weighted averaged fluorescence lifetime of FUL with a direct excitation is equal to 0.545 ns. The excitation of fullerenol complexes with ADH or HSA in the protein band results in an approximately three-fold increase in the lifetime of FUL, which proves the energy transfer from the protein to FUL.

## 3. Discussion

In a series of methods concerning the interaction of drugs, drug-carriers or xenobiotics, and protein, fluorescence techniques are great aids in the study of interactions between molecules and serum albumin because of their high sensitivity, rapidity, and ease of implementation [51,52]. The aim of this investigation was to study the affinity of fullerenol C_60_(OH)_36_ for ADH and HSA using UV-visible and fluorescence spectroscopy to understand the carrier role of serum albumin for such a compound in the blood under physiological conditions. Significantly, the determination and understanding of the putative drug carrier as FUL interacting with serum albumin is important for the therapeutic potential and design of drugs.

In plasma, HSA accounts for over 55% of the protein. The basic physiological function of albumin is to bind water, thanks to which the appropriate oncotic plasma pressure and its correct volume are maintained. HSA transports fatty acids, hormones, vitamins, metabolites, and some drugs. ADH was selected for this research as a protein with a function different from has, which is not subjected to functional change under the influence of FUL. It should be added that the concentrations of FUL used in the study did not affect the enzymatic activity of ADH. Our earlier studies also did not show the effect of FUL on ADH enzymatic activity even after a 24-h incubation [53].

Based on the data compiled in Table 2 and the absorption spectra of fullerenol in the presence of proteins (Figure 3), it can be seen that ADH and HSA did not change the fullerenol absorption coefficient regardless of its concentration. This suggests the lack of aggregation or dissociation of fullerenol nanoparticles in the presence of both proteins. This is an interesting observation, because fullerenol shows a tendency to aggregate in aqueous solutions, which can be seen from the NTA studies (Table 1, Figure 1) and the reports of other authors [52,54]. Prevention of the aggregation of FUL by proteins and binding to HSA protein may perform a role in the use of these nanoparticles as drug transporters. Drug transporters are developed to efficiently deliver the required amount of drugs to the appropriate destinations and maintain the desired drug concentration. Among others, liposomes, niosomes, nanoparticles, and polymers are used as drug transporters. Fluorescence techniques are a common method for studying the binding properties of small molecules to protein. Generally, the intrinsic fluorescence of protein is caused by tryptophan (Trp), tyrosine (Tyr), and phenylalanine (Phe) residues. The microenvironment of a fluorophore and the intrinsic fluorescence intensity of protein may change when the FUL interacts with ADH and HSA [54]. In this study, the interactions between the FUL and ADH and HSA were investigated by measuring the intrinsic fluorescence intensities of protein in the absence and presence of FUL. For analytical purposes, tryptophan fluorescence and energy transfer between tyrosine and tryptophan were used. When ADH and HSA were mixed with varying amounts of FUL, the fluorescence signal decreased. Moreover, the quenching was stronger in the case of HSA. The assessment of the tryptophan fluorescence quenching of ADH and HSA by FUL indicates that in both proteins, fluorescence is effectively quenched by FUL, both during the excitation of Tyr and Trp together (λ_ex_ = 280 nm) and only Trp (λ_ex_ = 295 nm).

An important issue to address was the occurrence of fullerenol absorption in the excitation bands of proteins (280 nm and 295 nm). For this reason, the measurement data were corrected on the inner filter effect, which eliminated the influence of fullerenol absorption on the amount of energy reaching protein fluorophores [41,42].

It should be added that FUL in the concentration range used in our experiments has a constant molar absorption coefficient at both wavelengths used for protein excitation.

The deviation of the Stern-Volmer plots from linearity (upward curvature) constructed from steady-state fluorescence intensity data pointed to the simultaneous existence of several types of interactions within proteins. The Stern-Volmer plots demonstrated both dynamic and static quenching components. However, FUL as the quencher and the fluorophore, i.e., protein, does not form a non-fluorescent complex during the static quenching but is in such close proximity that fluorescence is quenched immediately upon excitation. This mechanism is called sphere-of-action quenching.

The Stern-Volmer constants for dynamic quenching (K_SV_) and the constant for static quenching (V) indicate that an energy transfer occurs much more efficiently between HSA and FUL than between ADH and FUL. As can be seen in Table 5, the binding constant K_b_ for HSA:FUL is several orders of magnitude higher than for ADH:FUL, suggesting a high binding affinity between serum albumin and fullerenol. In the case of HSA protein, saturation by fullerenol was observed. The number of binding sites calculated for HSA is 2, i.e., two fullerenol molecules are bound to one molecule of HSA.

The fluorescence lifetimes (ex. 280 nm/em. 340 nm) for ADH and HSA are 2.6894 ns and 3.9624 ns, respectively. When the FUL received energy from a donor (protein) with a much longer lifetime, its mean lifetime obtained with a detection of 470 nm becomes longer. This situation is caused by the fact that the excitation of the FUL takes place continuously by an energy transfer from the protein to FUL, and not as a result of very short laser pulses, thanks to which the lifetime of the FUL is approximately three-fold longer. The occurrence of energy transfer from the protein to FUL, as evidenced by the longer fluorescence lifetime of the FUL, confirms the formation of the protein-FUL complex. The obtained results correspond very well with the theory and results presented in [55,56].

The obtained results indicate that both studied proteins, despite their structural and functional differences, interact with the FUL. However, the interaction of the FUL with HSA is much stronger, i.e., 3 orders of magnitude, than with ADH. All the experimental results clarify that FUL can tightly bind to HSA. Moreover, the binding of FUL to ADH did not affect the enzymatic activity of this protein, as was shown in a previous study [53]. FUL could bind to plasma albumin and be transported by it. At the same time, the enzymatic function of ADH is preserved, and the binding of FUL to ADH is much weaker.

The obtained results may be of practical importance in biological sciences in terms of the use of FUL as a drug carrier or a contrast agent in bioimaging [57,58], because the binding of drugs by albumin plays a key role in the pharmacokinetics of many drugs and their distribution in the body. Albumin is a natural carrier of many drugs, and knowledge on the binding ability of FUL to HSA may define new pathways in biomedicine.

## 4. Materials and Methods

### 4.1. Reagents and Materials

Pristine fullerene (C_60_) with manufacturer-declared 99.5% purity was obtained from SES Research (SES Research, Houston, TX, USA). Fullerenol (C_60_(OH)_36_) was synthesized from C_60_ (99.5%), sodium hydroxide, methanol, and hydrogen peroxide 30% (Avantor Performance Materials Poland SA, Gliwice, Poland) and Amberlit MB20 (Sigma-Aldrich, St. Louis, MO, USA). Human serum albumin (HSA, EC 274-272-6), alcohol dehydrogenase (ADH, EC 1.1.1.1), and phosphate-buffered saline (PBS) (pH 7.4) were obtained from Merck KGaA, Darmstadt, Germany. All solutions were prepared using ultra-purified water (Milli-Q Plus system).

### 4.2. Methods

In this study, the ability of fullerenol C_60_(OH)_36_ to bind to and to interact with proteins, i.e., alcohol dehydrogenase from *Saccharomyces cerevisiae* (ADH) and human serum albumin (HSA), were examined. Fluorescence spectroscopy, fluorescence quenching titration, and fluorescence lifetime measurement were used in this study. Nanoparticle size and concentration in different solutions were measured and visualized with nanoparticle tracking analysis (NTA).

#### 4.2.1. Synthesis of Fullerenol Nanoparticles

Fullerenol C_60_(OH)_36_ was synthesized from fullerene C_60_ (99.5%). Ingredients needed for the synthesis of C_60_(OH)_36_ were sodium hydroxide and hydrogen peroxide 30%, methanol, and Amberlit MB20. The procedure of the fullerenol solution synthesis was described earlier by Krokosz et al. [53].

#### 4.2.2. Nanoparticle Tracking Analysis

The measurement size of fullerenol (8 μM) was conducted using the nanoparticle tracking analysis (NTA). NTA is a technique used to detect polydisperse nanosized particles. The principle of the method consists of the visualization of the light scattered by individual nanoparticles moving under the Brownian motion. Through the application of the Stokes-Einstein equation, particle size and concentration can be calculated. NTA measurements were performed with a NanoSight NS300 equipped with a Blue488 laser type (Malvern Panalytical Ltd, Malvern, UK). The samples were measured at 22.6 °C in water and 0.02 M phosphate buffer at pH 7.4.

#### 4.2.3. ζ-Potential Measurements

The measurement of zeta potential was conducted using Zetasizer Nano-ZS, Malvern Instruments, Malvern, UK. Zeta potential values were calculated directly from the Helmholtz-Smoluchowski equation using the Malvern software (Malvern Instruments, Malvern, UK). The sample preparation procedure was described earlier by Lichota et al. [59].

#### 4.2.4. Preparation of the Sample of Fullerenol with Proteins

The C_60_(OH)_36_ suspension of 20–200 μL (stock 800 µM) was added to 100 μL of protein (ADH or HSA) at the concentration of 60 μM and adjusted to 3 mL with a 0.02 M phosphate buffer (pH = 7.4). The final concentration of proteins was 2 μM. The change in the concentration of fullerenol in the samples ranged between 2.64 and 133.36 μM. Each sample was prepared separately.

#### 4.2.5. Spectroscopic Measurements

All the measurements were carried out at 25 °C. The study evaluated the change in the UV absorbance, fluorescence, and fluorescence lifetime. The UV-Vis absorption spectra for proteins with fullerenol were measured in the range of 260–300 nm on a spectrophotometer (Varian Cary 5000 UV-Vis, Agilent Technologies, Santa Clara, CA, USA). Fluorescence emission spectra were measured with a spectrofluorometer (Varian Cary Eclipse Fluorescence Spectrophotometer, Agilent Technologies, Santa Clara, CA, USA). All measurements were conducted in a cuvette with an optical path length of 10 mm, corrected on the inner filter effect type I caused by the absorption of excitation light by FUL and protein. The inner filter effect of type II was not found under the experimental conditions. The corrections of fluorescence were carried out in accordance with the methodology described in the paper [41,42].

#### 4.2.6. Lifetime Measurements

The fluorescence excited-state lifetimes of fullerenol, ADH, and HSA with and without C_60_OH_36_ were measured using a FluoTime 200 lifetime fluorometer (PicoQuant, GmbH, Berlin, Germany) equipped with an R3809U-50 microchannel plate photomultiplier (MCP-PMT, Hamamatsu, Hamamatsu City, Japan) and a PicoHarp300 TCSPC module (PicoQuant, GmbH, Berlin, Germany). All the samples were measured at a temperature of 25 °C. The fluorescence lifetimes were calculated using the FluoFit software package (version 4.4)—PicoQuant, GmbH, Berlin, Germany.

## 5. Conclusions

Drug transporters are developed to efficiently deliver the required amount of drugs to the appropriate destinations and maintain the desired drug concentration. Human serum albumin (HSA) transports many endogenous and exogenous compounds and is responsible for osmotic blood pressure. The binding of compounds to HSA depends on many factors such as solubility, toxicity, susceptibility to oxidation, in vivo half-life, etc. In this study, the binding of fullerenol C_60_(OH)_36_ to two structurally and functionally different proteins was confirmed. The binding constant K_b_ for HSA and the FUL complex was several orders of magnitude higher than for ADH and the FUL complex. Moreover, protein saturation by fullerenol was observed for HSA and the number of binding sites n obtained for HSA was equal to 2. For ADH, protein saturation with fullerenol was not achieved.

The presence of protein in the fullerenol solution prevents FUL aggregation. Prevention of the aggregation of fullerenol by proteins and binding to HSA may perform a role in the use of these nanoparticles as drug transporters.

The results from the steady state and time-resolved measurements indicated that both quenching mechanisms, dynamic and static, are present in the studied proteins quenched by the FUL. In addition, from Stern-Volmer plots, the presence of sphere-of-action quenching is suggested. This effect is more pronounced for HSA.

The transfer of energy from the protein to FUL was also proved. The Stern-Volmer constants for dynamic quenching (K_SV_) and the constant for static quenching (V) indicate that an energy transfer occurs much more efficiently between HSA and FUL than between ADH and FUL.

Changes in the lifetimes confirm the existence of the dynamic quenching mechanism. Stern-Volmer plots for the amplitude-weighted average lifetimes of proteins in the absence and the presence of FUL resulted in a linear plot for ADH. Unlike ADH, the Stern-Volmer plot for HSA was nonlinear. The downward curve suggested the presence of additional processes between HSA and the FUL in the excited state.

In summary, both studied proteins, despite their structural and functional differences, interact with FUL. However, the interaction of FUL with HSA is much stronger, i.e., 3 orders of magnitude, than with ADH. The obtained results may be of practical importance in biological sciences in terms of the use of FUL as a drug carrier or a contrast agent in bioimaging.

## Figures and Tables

**Table 1:** Mean size and concentration of C_60_(OH)_36_ from NTA and zeta potential measurements.

Parameter	Water	0.02 M Phosphate Buffer pH 7.4
Mean (nm) ± SD	186.5 ± 76.6	164.4 ± 65.6
Mode (nm)	132.8	117.3
Concentration (particles/mL)	2.65 e + 008	4.55 e + 008
Zeta potential (mV) ± SD	−27.5 ± 1.0	−37.4 ± 3.0

**Table 2:** Molar absorption coefficients of ADH, HSA, and fullerenol.

	Molar Extinction Coefficient ε_280_ [mol^−^^1^ l cm^−^^1^]
ADH	167,900
HSA	35,340
FUL in 0.02 M phosphate buffer pH 7.4	43,336

**Table 3:** Fluorescence lifetime (τ_i_), pre-exponential factor (α_i_), amplitude-weighted average fluorescence lifetime <τ>, and quality of fit (${Χ}_{R}^{2}$) for ADH and HSA titrations with FUL.

	Concentration of FUL [µM]	τ_1_ [ns] (α_1_)	τ_2_ [ns] (α_2_)	τ_3_ [ns] (α_3_)	<τ> [ns]	${Χ}_{R}^{2}$
**ADH + FUL**	0	4.8040	1.8636	0.5017	2.6894	0.998
		(0.3909)	(0.3716)	(0.2376)		
	2.64	4.8590	2.0210	0.6145	2.7143	0.969
		(0.3750)	(0.2611)	(0.2638)		
	5.36	4.7977	1.8792	0.5522	2.6606	0.987
		(0.3793)	(0.3753)	(0.2454)		
	13.36	4.8332	1.9843	0.5832	2.6684	0.972
		(0.3709)	(0.3631)	(0.2660)		
	26.64	4.8140	1.9055	0.5346	2.6182	0.986
		(0.3669)	(0.3745)	(0.2586)		
	40.00	4.8168	1.9429	0.5514	2.6397	0.989
		(0.3703)	(0.3658)	(0.2640)		
	53.36	4.8025	1.9342	0.5385	2.6075	1.018
		(0.3654)	(0.3662)	(0.2684)		
	80.00	4.8393	2.0225	0.6241	2.6221	0.972
		(0.3518)	(0.3683)	(0.2798)		
	106.64	4.8105	1.9846	0.5850	2.5839	0.994
		(0.3507)	(0.3694)	(0.2799)		
	133.36	4.7757	1.9377	0.5571	2.5306	0.987
		(0.3495)	(0.3617)	(0.2889		
**HSA + FUL**	0	7.2305	3.4098	0.8234	3.9624	1.010
		(0.3359)	(0.3815)	(0.2826)		
	2.64	7.2217	3.3001	0.7711	3.7433	1.007
		(0.3175)	(0.3653)	(0.3171)		
	13.36	7.0044	2.8965	0.6909	3.4167	1.009
		(0.3093)	(0.3504)	(0.3403)		
	40.00	6.9017	2.6751	0.6620	2.9743	0.982
		(0.2574)	(0.3509)	(0.3918)		
	80.00	6.7892	2.5679	0.6879	2.6013	0.970
		(0.2047)	(0.3534)	(0.4419)		
	133.36	6.5247	2.3054	0.6084	2.2367	0.963
		(0.1720)	(0.3600)	(0.4681)		

**Table 4:** The calculated parameters for ADH and HSA fluorescence quenching by the FUL.

TIME-RESOLVED MEASUREMENTS				
		Dynamic Quenching		
	***λ_ex_/λ_em_*** **[nm] **	***K_SV_*** **[M^−1^] **		***R* ^2^**
** ADH + FUL **	280/330	461 ± 43		0.9999
** HSA + FUL **	280/340	10869 ± 989		0.9998
**STEADY-STATE MEASUREMENTS **				
		** Dynamic Quenching **	** Static Quenching **	
	*** λ_ex_/λ_em_*** **[nm] **	***K_SV_*** **[M^−1^] **	***V*** **[M^−1^] **	***R* ^2^**
** ADH + FUL **	280/304	461	8796 ± 68	0.9977
	280/340	461	4917 ± 103	0.9887
	295/340	461	4610 ± 104	0.9872
** HSA + FUL **	280/304	12353 ± 1919	14067 ± 753	0.9993
	280/340	13097 ± 1528	11742 ± 583	0.9994
	295/340	10831 ± 1956	10966 ± 837	0.9988

**Table 5:** The binding parameters of the ADH-FUL and the HSA-FUL complex at different concentrations of FUL.

	λ_ex_/λ_em_ [nm]	Concentration of FUL [M]	K_b_ [M^−1^]	n	R^2^
** ADH + FUL **	280/304	2.64 × 10^−6^–1.40 × 10^−5^	136.6	0.60 ± 0.03	0.9957
	280/340		17.5	0.45 ± 0.04	0.9789
	295/340		3616	0.91 ± 0.17	0.9663
	280/304	2.66 × 10^−5^–5.34 × 10^−5^	1.25 × 10^5^	1.24 ± 0.04	0.9992
	280/340		1.19 × 10^5^	1.30 ± 0.15	0.9869
	295/340		1.36 × 10^5^	1.31 ± 0.10	0.9941
	280/304	8.00 × 10^−5^–1.33 × 10^−4^	8.00 × 10^6^	1.68 ± 0.11	0.9956
	280/340		1.82 × 10^6^	1.61 ± 0.06	0.9985
	295/340		1.95 × 10^6^	1.62 ± 0.03	0.9997
** HSA + FUL **	280/304	2.64 × 10^−6^–1.40 × 10^−5^	8671	0.87 ± 0.02	0.9993
	280/340		6246	0.84 ± 0.01	0.9999
	295/340		3896	0.81 ± 0.03	0.9990
	280/304	1.40 × 10^−5^–4.00 × 10^−5^	2.02 × 10^5^	1.15 ± 0.02	0.9998
	280/340		6.23 × 10^4^	1.05 ± 0.01	0.9999
	295/340		6.34 × 10^4^	1.06 ± 0.04	0.9987
	280/304	8.00 × 10^−5^–1.33 × 10^−4^	2.72 × 10^10^	2.38 ± 0.02	0.9999
	280/340		2.02 × 10^9^	2.12 ± 0.06	0.9992
	295/340		1.20 × 10^9^	2.09 ± 0.01	0.9999

**Table 6:** The fluorescence lifetimes of FUL observed at 470 nm. The excitation wavelength was 375 nm for FUL in the phosphate buffer and 280 nm for the HSA and ADH complexes (the FUL concentration was 14 µM).

	τ_1_ [ns](α_3_)	τ_2_ [ns](α_1_)	τ_3_ [ns](α_2_)	τ_4_ [ns](α_3_)	<τ>[ns]	${Χ}_{R}^{2}$
** FUL in 0.02 M Phosphate Buffer pH 7.4 **	6.4423	1.7801	0.3502	0.0531	0.545	1.040
	(0.0285)	(0.1304)	(0.2860)	(0.5552)		
** ADH + FUL **	6.3530	2.5694	0.9725	0.2078	1.533	0.960
	(0.0855)	(0.2614)	(0.2389)	(0.4142)		
** HSA + FUL **	8.667	3.061	1.1289	0.3005	1.637	0.959
	(0.0658)	(0.1869)	(0.3260)	(0.4212)		

## Data Availability

Not applicable.
